# The Roles of Glycans in Bladder Cancer

**DOI:** 10.3389/fonc.2020.00957

**Published:** 2020-06-12

**Authors:** Yuli Jian, Zhongyang Xu, Chunyan Xu, Lin Zhang, Xiaoxin Sun, Deyong Yang, Shujing Wang

**Affiliations:** ^1^Department of Biochemistry and Molecular Biology, Institute of Glycobiology, Dalian Medical University, Dalian, China; ^2^Department of Urology, First Affiliated Hospital of Dalian Medical University, Dalian, China

**Keywords:** glycans, glycosyltransferases, bladder cancer, abnormal glycosylation, biomarkers

## Abstract

Bladder cancer is one of the most common malignant tumors of the urogenital system with high morbidity and mortality worldwide. Early diagnosis and personalized treatment are the keys to successful bladder cancer treatment. Due to high postoperative recurrence rates and poor prognosis, it is urgent to find suitable therapeutic targets and biomarkers. Glycans are one of the four biological macromolecules in the cells of an organism, along with proteins, nucleic acids, and lipids. Glycans play important roles in nascent peptide chain folding, protein processing, and translation, cell-to-cell adhesion, receptor-ligand recognition, and binding and cell signaling. Glycans are mainly divided into N-glycans, O-glycans, proteoglycans, and glycosphingolipids. The focus of this review is the discussion of glycans related to bladder cancer. Additionally, this review also addresses the clinical value of glycans in the diagnosis and treatment of bladder cancer. Abnormal glycans are likely to be potential biomarkers for bladder cancer.

## Introduction

Bladder cancer is a malignant tumor of the urogenital system, which can be divided into bladder urothelial carcinoma, bladder squamous cell carcinoma, bladder adenocarcinoma, small cell carcinoma of bladder, and so on. The most common type of bladder cancer is bladder urothelial carcinoma. Smoking is considered to be the main risk factor of bladder cancer, and its incidence increases with age. There are significant gender differences in the incidence and prognosis of bladder cancer have obvious gender differences. The incidence of bladder cancer ranks fourth and eleventh among men and women with cancer, respectively (1). Early diagnosis and specific treatments are effective means of treating bladder cancer (2). Great progress has been made in the study of bladder cancer in various fields, but there are still has problems such as poor prognosis, serious complications, and high recurrence rate (3). Therefore, searching for specific molecular targets and treatment methods for bladder cancer is a hot spot of current research.

Polysaccharides are one of the basic components of animal and plant cells. They can be divided into N-glycans, O-glycans, glycosaminoglycans, and glycosphingolipids according to the different ways of combining sugar chains. Glycans play an important role in cell-to-cell interactions and signal transduction. Abnormal sugar chains often play important roles in cancer biology as tumor markers. Glycans bind to proteins, lipids, and other glycosyl groups through glycosylation, a common post-translational modification process (4). As a key regulatory mechanism, glycosylation regulates the physiological, and pathological processes of some cells. Abnormal glycosylation is a common phenomenon that occurs in cancer cells, and it can determines the stage, direction, and fate of tumor progression (5).

Compared with normal tissues, there are many changes in glycans in cancer cells, including fucosylation, sialylation, truncation of O-glycans, and increased branching of O-glycans (6). Abnormal glycoproteins are involved in regulating tumor cell proliferation and adhesion-related signaling pathways. The main cause for these phenomena is abnormal overexpression of glycosyltransferases or glycosidases (7). Glycans on the cell surface can be used as targets for the treatment of cancer, but due to heterogeneity and complexity of the sugar chain structure, it is still a problem for anaylzing the detailed sugar chain structure (8). This review summarizes the research progress of some abnormal glycans in bladder cancer, and details their biological functions in bladder cancer according to the types of glycans.

## N-glycan

N-glycans are classified into high mannose type, complex type, and heterozygous type according to their structure. They all have a common pentasaccharide core, and their synthesis is catalyzed by specific glycosyltransferase or glycosidase. Abnormal glycosylation catalyzed by glycosyltransferases and glycosidases is often closely related to tumorigenesis, development, and metastasis (9). Glycosylation is one of the most common post-translational modifications of proteins. It is a process of transferring glycosyl group to the specific amino acid residues on proteins to form glycosidic bonds under the action of glycosyltransferase. In human tumors, cell surface growth factors and lectins play important roles in the transformation and development of tumors (10, 11). Studies have shown that when epithelial-mesenchymal transition (EMT) occurs in non-malignant bladder transitional epithelial HCV29 cells induced by transforming growth factor-β (TGFβ), the expression of hybrid-type glycans increases, while the expression of complex-type glycans decreases. Transcription of the *fuca1* gene encoding Type 1 α-L-fucosidase was suppressed in bladder cells with EMT, which led to increased levels of fucosylated N-glycans (12). In addition, the change of specific N-glycans on the cell surface combined with EMT contributes to cell migration (13). This indicates that when EMT occurs in bladder cells, the levels of N-glycosylation changes, which in turn promotes tumor proliferation and metastasis. Therefore, further discovering and studying of the changes in the structure and function of N-glycans related to bladder tumors can better assess the development of bladder cancer, which will have important significance for the diagnosis, treatment, and prognosis of bladder cancer.

### Fucosylation

Fucosylation is a process in which GDP-fucose is used as a donor to transfer glycosyl to proteins or lipids under the catalysis of fucosyltransferase, which is often involved in cell differentiation, development, and malignant transformation. According to the location of fucose, fucosylation can be divided into core fucosylation (α-1,6 fucosylation) and terminal fucosylation (α-1,2 and α-1,3/4 Fucosylation). There are currently 13 known fucosyltransferases involved in fucosylation, of which fut8 is the only transferase that catalyzes core fucosylation, fut1, and fut2 are involved in α1–2 linked fucose synthesis, fut3–9 participate in the synthesis of α1–3 and α1–4 linked fucose (14). Calreticulin can regulate the content of Fut1 in bladder cancer tissue. Modification of β1 integrin with α1,2 fucosylation can regulate cell adhesion and metastasis of bladder cancer cells when the expression levels of fut1 were upregulated (15). In tumor tissue, overexpression of fut4 transferring GDP-fucose to the Lewis Y antibody terminal N-GlcNac with the 1,3-linkage, which promoting neoplastic cell proliferation (16). MiR-125a-5p can inhibits cell proliferation and induce apoptosis, and reverse the EMT process of bladder cancer cells by targeting fut4, thereby, inhibiting tumor cell metastasis (17). Studies have found that expression levels of complex fucosylated N-glycan was abnormal in bladder cancer tissues (including core fucosylated N-glycans levels increased and terminal fucosylated N-glycan levels decreased), and the core fucose expression level was positively correlated with tumor tissue grade (18). Therefore, changes in intracellular fucose levels may be closely related to the progress of bladder cancer, but the specific molecular mechanisms need to be further explored.

### Sialylation

Sialic acid is a nine-carbon monosaccharide with negatively charge, and exists on the surface of cells and the outermost ends of most vertebrate glycoproteins and glycolipid molecules. It participates in molecular recognition and adhesion processes, and it is an important information transfer molecule in the organisms. Free sialic acid is catalyzed by CMP-Sia synthase in the presence of CTP to generate donor CMP-Sia. Under the catalysis of sialyltransferase, donor CMP-Sia is attached to the sugar complex (N-glycans, O-glycans, and glycolipids) via a α2,3, α2,6, α2,8 linkage.

Abnormal glycosylation can often be found in tumor cells. One of the important changes is the alteration of sialylated glycans. The appearance of abnormal sialylated glycans is often accompanied by tumor occurrence, development, invasion, and metastasis. Abnormal sialylation is regulated by sialyltransferase and sialidase levels. Glycans related to human bladder cancer have been discovered as follows. The blood group antigen Lewis X (LeX) has been considered as a biomarker for urothelial cancer. It is usually not found in normal urothelial cells in adults, but is expressed in transitional cell tumors, and has nothing to do with the stage and grade of the tumor (19). α-2,3-linked sialyltransferases ST3Gal III, ST3Gal IV, and ST3Gal VI are key enzymes that mediate sialyl Lewis A and sialyl Lewis X synthesis. Sialyl Lewis A (sLeA, also known as CA19-9) and sialyl Lewis X (sLeX) play important roles in cancer progression. The clinical usefulness of monitoring CA19-9 in urothelial carcinoma is less commonly described. Monitoring the level of CA19-9 in urine may help diagnose bladder urothelial carcinoma (20). On the other hand, serum CA19-9 is almost always elevated in metastatic and spread tumors (21). However, it comes into view that the role of CA19-9 as a serum marker in urothelial cancers has not yet been defined. SLeX plays a key role in the selection of selectins and is involved in the spread of tumor cells (22). It is reported that the expression of sialylated Lewis X antigen is closely related to the invasiveness and metastatic potential of bladder cancer (23). Another sialylated glycan in bladder cancer is sTn, which is usually produced by overexpression of ST6GALNAC1 sialyltransferase. ST6GALNAC1 transfers sialic acid to the O atom at the sixth position of the N-acetylgalactosamine residue linked to a serine or threonine (GalNAc α-O-Ser/Thr) on specific proteins, thereby preventing extension of O-glycosidic chains in glycoproteins. Therefore, sTn is a truncated abnormal O-glycan, which often appears in tumor mucin. In high grade bladder cancer tissues, the significant overexpression of sTn antigen is associated with increased expression of ST6GALNAC1 sialyltransferase and down-regulates anti-cancer immune response through different mechanisms (24). Generally, polysialic acid (PSA) is formed when sialic acid is linked to another sialic acid residue through an α-2,8 glycosidic bond. PSA can regulate the interactions between cells and cells, cells and cell matrix, and cell surface molecules. PSA can promote cancer metastasis and participate in nervous system development. In summary, the change in the content of sialylated glycans is directly related to the development of bladder cancer, and the sialyltransferases that catalyze the production of glycans play very important roles in it, which provides clues for us to understand the mechanism of tumor development.

### β1–6-Branching GlcNAc

**Figure 1:**
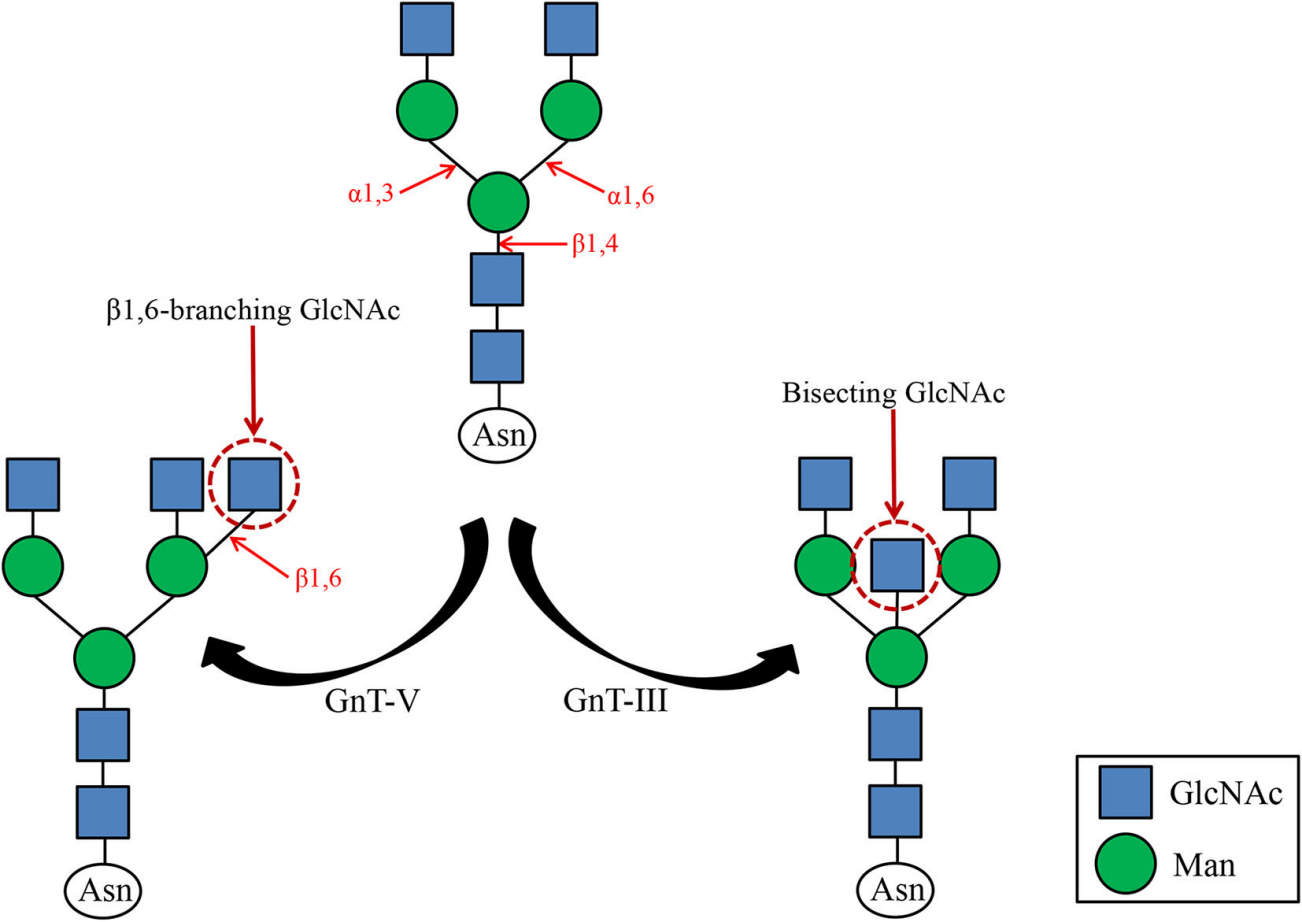
GnT-III and GnT-V catalyzed glycosylation reactions. GnT-III catalyzes the formation of bisecting GlcNAc and regulates tumor cell metastasis. GnT-V catalyzes the formation of β1,6 branched N-glycans, a structure commonly found in tumor cells.

N-acetylglucosaminotransferase V (GnT-V) alters the structure of specific N-glycans by modifying α1–6-linked mannose with the β1–6-linked N-acetylglucosamine (Figure 1). Increased β1–6 GlcNAc branch expression was observed in many cancers, and was closely related to malignant tumor metastasis and poor prognosis (25, 26). The formation of β1–6 branches on cell surface receptors accelerates cancer metastasis (27). And GnT-V expression is related to tumor stage or grade. The main reason affecting this biological behavior may be that GnT-V can affect downstream molecular signal transduction by changing the β1–6 branches of certain glycoproteins on the cell surface, such as cadherin, integrin, and cell surface growth factor receptors (GFR) (28, 29). It has been reported that in superficial bladder cancer, the expression of GnT-V is detected by immunohistochemistry, showing that its expression negatively correlated with tumor grade and stage (30). The incidence of GnT-V positive expression in bladder cancer is significantly higher in lower grade/less invasive cancer than in higher grade/higher invasive cancer, which indicates that immunohistochemical detection of GnT-V can be used as a reliable marker for predicting the recurrence of superficial bladder cancer. Therefore, we concluded that GnT-V and its catalytically formed β1–6GlcNAc branch N-glycan are closely related to tumor cell adhesion, invasion, and metastasis. Together, better understanding the relationship between GnT-V and tumor stage, grade, and prognosis will help clinical early diagnosis of the disease and evaluation of treatment effects.

### Bisecting GlcNAc

N-acetylglucosamine transferase III (GnT-III) transfers N-acetylglucosamine (GlcNAc) from UDP-GlcNAc to the core mannose of N-glycan to form a β1,4 glycosidic bond, forming a bisecting GlcNAc (Figure 1). Since GnT-III and GnT-V act on the same substrate, and the bisecting GlcNAc prevents GnT-V from approaching the dual antenna substrate, it is speculated that GnT-III is an antagonist of GnT-V. For the same target protein integrin α3β1, opposite effects of GnT-III and GnT-V have been observed (31). GnT-V promotes α3β1 integrin-mediated cell migration, while GnT-III overexpression inhibits GnT-V-induced cell migration, proving that GnT-III and GnT-V can competitively modify the same glycoprotein, thereby further regulate their biological functions positively or negatively. Previous studies have shown that the expression of GnT-III and the emergence of GlcNAc can promote tumor migration and invasion, such as ovarian cancer (32). However, in some tumors, E-cadherin can up-regulate GnT-III level, forming a positive feedback loop between the two to promote cell adhesion (33). The reason is that GnT-III overexpression makes the retention time of E-cadherin on the cell surface prolonged, which leads to enhanced intercellular adhesion. Therefore, overexpression of GlcNAc can suppress tumor metastasis. It can be seen that GnT-III catalyzes the formation of bisecting GlcNAc and no longer forms branches, which affects the function of cell surface adhesion molecules and then the biological function of tumors. It has been reported that the structure and content of N-glycans on urinary fibronectin (Fn) in bladder cancer patients have changed significantly, especially the increase in bisecting GlcNAc content (34). However, there are few studies about the effect of bisecting GlcNAc on the biological behavior of bladder cancer, and the specific molecular mechanism needs to be further explored.

## O-glycan

There are different core structures between O-glycans and N-glycans, but biologically active oligosaccharides are usually found on the outer chain attached to the core. Previous studies have found that O-glycan is involved in tumor metastasis. Interestingly, O-glycans show dual roles in tumor metastasis depending on their structure (35). The lack of the core 2 structure on human leukocyte antigen class I (HLA class I) O-glycans and the impairment of galectin-glycan lattice formation allow bladder cancer cells to escape cytotoxic T lymphocytes (CLTs) anti-tumor immunity (36). Mucin 1 (MUC1) on the surface of bladder cancer cells carrying core 2 O-glycans acts as a molecular barrier to resist the attack of natural killer (NK) cells, thereby promoting bladder tumor metastasis (37). Core 2 O-glycans is catalyzed by β-1,6-N-acetylglucosaminyltransferase (core 2 β-1,6 GlcNAc transferase or C2GnT), and found that C2GnT expression is positively correlated with urinary system tumors such as bladder cancer (38, 39). These results suggest that C2GnT expression promotes tumor metastasis. In contrast to C2GnT, core 3 O-glycan catalyzed by Core 3 synthase inhibits tumor metastasis by regulating integrin-mediated signaling (40). In summary, O-glycans with different structures play different roles in tumor metastasis. Therefore, we should thoroughly explore the specific molecular mechanism of the effect of O-glycan structure and corresponding glycosyltransferase on bladder tumor metastasis.

### O-GalNAc Glycans

**Figure 2:**
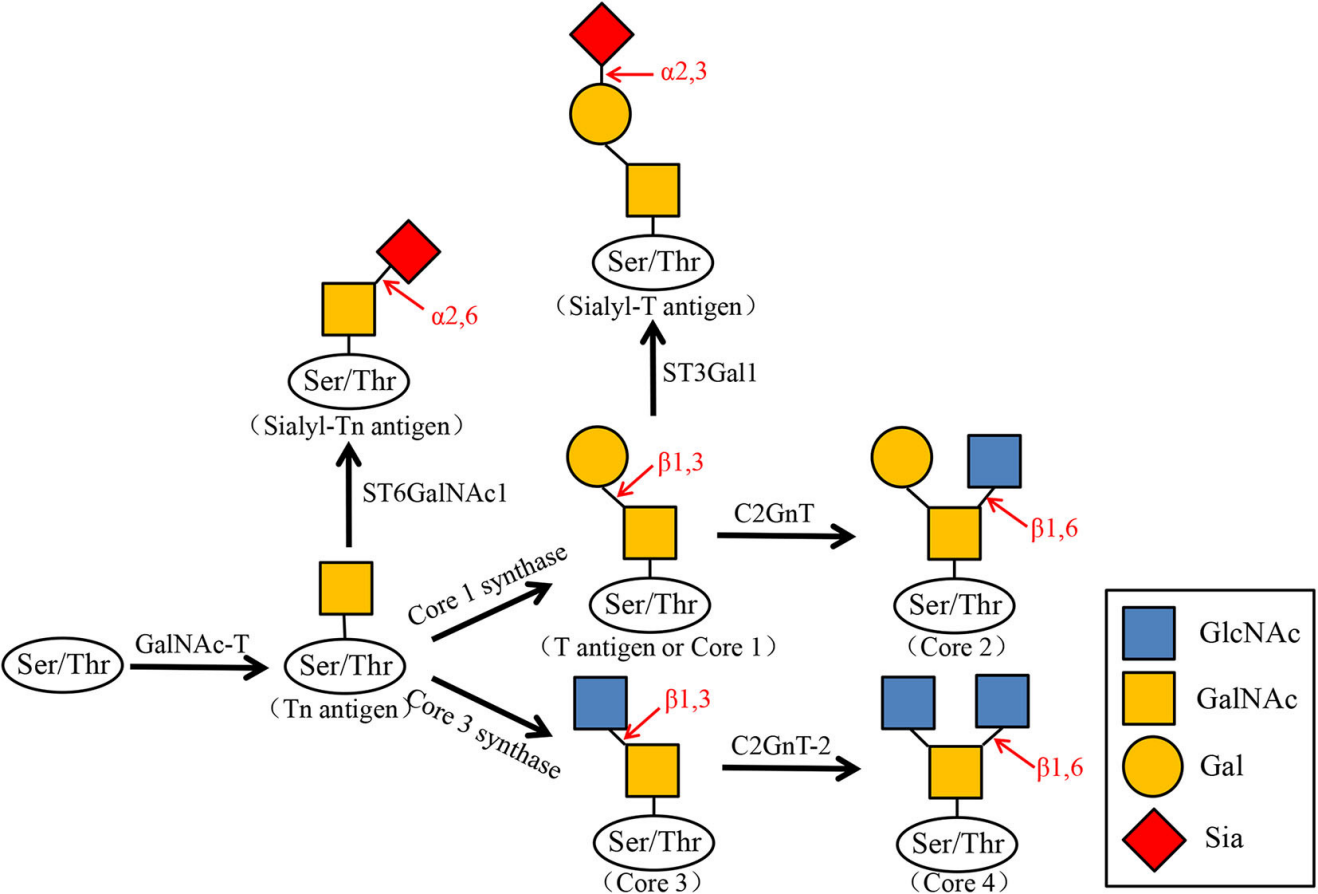
Biosynthesis of O-GalNAc glycans and their sialylated glycans. N-acetylgalactosamine (GalNAc) is transferred to the serine (Ser) or threonine (Thr) residues of the polypeptide by GalNAc transferase (GalNAc-T). GalNAcα1-Ser/Thr (Tn antigen) is converted to Galβ1–3GalNAcα1-Ser/Thr (T antigen or Core1) by Core1 synthase. Core1 is then converted by C2GnT to Core2, and the Tn antigen can also be converted to Core3 by Core3 synthase. Core3 is converted by C2GnT-2 to Core4. At the same time, Tn antigen is transformed into Sialyl-Tn antigen by ST6GalNAc1, and Core1 is transformed into Sialyl-T antigen by ST3Gal1.

Many glycoproteins carry glycosyl groups induced by GalNAc, which are attached to the hydroxyl groups of serine or threonine residues. O-GalNAc glycans, also known as mucin-type O-glycans. The sugars found in O-GalNAc glycans include GalNAc, Gal, GlcNAc, Fuc, and Sia, while Man, Glc, or Xyl residues are absent. O-GalNAc glycans related to malignant tumors are generally incompletely synthesized O-glycans such as T antigen, Tn antigen, and their corresponding sialic acid variants (sT antigen and sTn antigen). The first step in the start of O-glycosylation is that N-acetylgalactosamine (GalNAc) is connected to the serine/threonine (Ser/Thr) residues of the polypeptide chain to form the Tn antigen. It is further added with galactose (Gal) to the three hydroxyl position to expand to core type 1 (T antigen), and then N-acetylglucosamine (GlcNAc) is added to the three or six hydroxyl position to form core type II and type III, and then sialylated as terminal modification, forming sTn antigen, sT antigen, and so on (Figure 2).

It has been reported that the T antigen system and mean nuclear volume have been proposed as risk variables for bladder tumors. There is a correlation between the mean nuclear volume and the expression of the T antigen system in bladder cancer tumors of different stages and grades, and the primary tumor high average nuclear volume is associated with tumor invasion (41). We can speculate the clinical course and invasion of the tumor by measuring the amount of the T antigen system. The appearance of sTn antigen is generally inextricably linked to the early occurrence of tumors, and it is expressed in bladder cancer (42), ovarian cancer (43), colon cancer (44), lung cancer (45), gastric cancer (46), and prostate cancer (47). An α2,6 sialyl acyltransferase ST6GALNAC1 was found to be overexpressed in bladder cancer tissues with higher malignancy, and this change is related to the sTn antigen, which is also explained above. Recently, a new anti-STn monoclonal antibody named L2A5 was developed. L2A5 can specifically bind to the sialylated structure on the surface of bladder cancer cell lines expressing STn, which can effectively target and block cancer-related antigens (48). It shows that antibody-based bladder cancer treatment has a good application prospect. Since elevated Tn, sialyl-Tn, and T antigen levels are associated with bladder cancer, O-GalNAc glycans can be used as potential markers for cancer diagnosis and prognosis.

### O-GlcNAcytion

O-GlcNac glycosylation is a progression in which GlcNac covalently bind to the ser/Thr hydroxyl group on a protein under the catalysis of OGT (O-linked GlcNAC transferase), and which can be eliminated by OGA (O-GlcNac glycosidase). O-GlcNac modification plays an important role in transcription regulation, signal transduction, and protein degradation (49). In addition, elevated O-GlcNac levels are closely related to tumor cell activity. For example, in tumor cells, overexpression of OGT adds O-GlcNac to Thr-352 of NF-κB p65, this process can decreases binding to IκBα, so that NF-κB can play its role of transcription factors through entering the nucleus, which leads to cell division defects and cloning (50). Bladder cancer cells prematurely terminate the O-glycosylation of proteins, allowing short-chain O-GlcNac glycans to accumulate on the cell surface, which can reduce the adhesion of tumor cells, thereby increasing their metastatic and invasive properties (51). Studies have found that OGT in urine is significantly different between early bladder cancer and advanced bladder cancer, so the content of OGT can be analyzed to classify bladder cancer (52). Chronic inflammation caused by some infection or anticancer drugs are important risk factors for bladder cancer (53), therefore, it can be concluded that abnormal O-GlcNac glycosylation may be related to the occurrence and development of bladder cancer. In summary, studies have shown that analysis of O-GlcNAcylation or OGT expression may be useful for the diagnosis of bladder cancer, and OGT may be used as a potential target for bladder cancer therapy in the future.

## Proteoglycan

Proteoglycans are composed of core proteins and glycosaminoglycans [GAG, for example, hyaluronic acid (HA), chondroitin sulfate (CS), dermatan sulfate (DS), heparin sulfate (HS), heparin (HP), and sulfuric acid Keratin (KS)] with covalent bonds. The structure of proteoglycan is different from glycoprotein. Proteoglycan is a key biologically active molecule that can directly affect tumor development and controls its development to varying degrees. Studies have revealed that the expression of Biglycan (BGN) is related to high grade human bladder tumor, and a series of experiments have proved that BGN is an inhibitor of bladder tumor cell growth and proliferation (54). Decorin is also a versatile small leucine-rich extracellular matrix proteoglycan that has been shown to have potent antitumor activity (55, 56). Studies have found that human bladder cancer cells do not express decorin *in vivo* or *in vitro*, and that decorin expression is reduced in malignant human bladder tissue samples (57). And the destruction of the *decorin* will not lead to the spontaneous development of the tumor, but the absence of the *decorin* will allow the tumor to form (58). Therefore, both biglycan and decorin have inhibitory effects, but they are regulated differently in bladder cancer, the mechanisms of blocking decorin expression in human bladder cancer cells need further research and clarification by scientists, and this will provide the possibility to detect decorin as a bladder cancer treatment tool.

Glycosaminoglycan (GAG), also known as mucopolysaccharide, aminopolysaccharide, and acid polysaccharide, is a repeating disaccharide unit composed of hexuronic acid and hexosamine. As mentioned above, there are six types of important glycosaminoglycans in the human body. GAG is involved in many important physiological and pathological processes, and plays a vital role in cell-cell interactions. The glycosaminoglycan layer present on the bladder surface can non-specifically prevent bacteria, ions, and molecules from adhering to the epithelium. In addition, studies have observed that chondroitin sulfate (CS) has a growth inhibitory effect on human bladder cancer cell line HT-1376, and the combined use with chemotherapy drugs CS, gemcitabine (GEM), or mitomycin-C (MMC) can make the cell growth inhibition more obvious, but the exact molecular mechanisms need to be further explored (59).

Hyaluronic acid (HA) is a non-sulfated glycosaminoglycan, a repeating unit consisting of disaccharides of D-glucuronic acid and N-acetylglucosamine (60). HA synthase (HAS1, HAS2, HAS3), HA receptor, and HYAL1 in the HA family play a role in tumorigenesis and development. HA can help tumor cells overcome contact inhibition and have protective effects on immune surveillance to a certain extent (61). Hyaluronic acid synthesis is regulated by glycogen debranching enzyme. We found that the loss of amylo-α-1–6-glucosidase-4-α-glucosyltransferase (AGL) drives rapid proliferation of bladder cancer cells by upregulating HA synthase (HAS2) mediated HA synthesis (62, 63). Studies have shown that the overexpresseion of HAS1 in bladder cancer cells can cause an increase in the CD44 variant isoforms (HA receptor), thereby inhibite Fas mediated apoptosis (64), and promoting the growth and invasion of bladder cancer cells. At the same time, the expression of CD44 is directly proportional to the aggressiveness of bladder tumor cells (65). HYAL1 and tumor-associated HA/HAase system can promote tumor growth, invasion and metastasis (66–68). The accuracy of HA and HAase in the diagnosis of bladder cancer is more than 90%, which is higher than that of HA and HAase alone. Therefore, HA/HAase can be used as a biomarker for diagnosis of bladder cancer (69, 70). It can be seen that the determination of HA/HAase is of great significance in the diagnosis and prognosis of bladder cancer (71), and the hyaluronic acid family can be targeted for cancer treatment.

## Glycosphingolipid

Glycosphingolipids (GSLs) are glycoside compounds where ceramide is glycosylated. In general, GSL was found to interact with key molecules in a specific membrane microdomain called a “glycosynapse,” mediating cell adhesion and signal transduction (72). Regarding how the sugar synaptic microdomains regulate the phenotype of tumor cells, some studies have shown that this is achieved by altering cell growth, adhesion, and movement (73). Sialic acid-containing acidic glycosphingolipids are called gangliosides. It is reported that gangliosides, especially GM2 and GM3, can interact with GFR and inhibit growth factor-induced signal transduction (74). Studies have shown that GM3 is highly expressed in human benign, non-invasive tumor KK47 cells, but GM3 levels are very low in highly malignant, invasive bladder cancer YTS1 cells. High GM3 levels reduce tumor cell motility/invasiveness, while low GM3 levels enhance tumor cell motility/invasiveness (75). These indicate the expression levels of GM3 are negatively correlated with the migration, invasion ability, and malignancy of bladder cancer cells. Therefore, it is of great significance to study the effect of GM3 expression on the biological behavior of bladder tumor cells.

## Conclusion and Perspectives

This article reviews the structure and function of glycans related to bladder cancer and the glycosyltransferases that catalyze glycan formation, and the roles of glycans on the biological functions of bladder cancer are summarized (Table 1). In recent years, people have paid more and more attention to the key role of glycans in cancer progression. The appearance of tumors is often accompanied by abnormal glycosylation, which determines the direction of tumor progression. Fucosylation and sialylation and other glycosylations affect the function of glycans, which is closely related to the abnormal expression of glycosyltransferase, or glycosidase. We know that the occurrence, development, proliferation, invasion, and metastasis of bladder cancer are inextricably linked to changes in glycosylation in cancer cells. However, due to the large differences in the molecular weight of the sugar chains formed by glycans, the complex linkages, and the immature technology of sequencing instrument analysis, the related molecular mechanisms need to be further explored. In addition, glycans are highly expressed in the body fluids of patients (such as serum and urine) and on the surface of cancer cells. We can use it as a marker for the daily diagnosis of cancer and provide assistance for tumor treatment and prognosis. Therefore, better understanding the changes of the structure and function of abnormal glycans in bladder cancer will be beneficial to the diagnosis and treatment of bladder cancer, and will provide the possibility for the development of new anti-tumor drugs.

**Table 1:** The roles of glycan components related to bladder cancer.

**Glycan components**	**Involved glycosyltransferases**	**Involved molecules**	**Impact on bladder cancer**	**References**
α1–2 fucose	FUT 1	β1 integrin	Promotion	(15)
SleA (CA19-9)/SLeX	ST3GAL III, ST3GAL IV, ST3GAL VI	Selectin	Promotion	(20–23)
STn	ST6GALNAC1	–	Promotion	(24)
β1–6-branching GlcNAc	GnT-V	Cadherin, integrin, and cell surface growth factor receptor	Suppression	(30)
Bisecting GlcNAc	GnT-III	–	To be studied	–
Core 2	β-1,6-N-acetylglucosaminyltransferase (C2GnT)	Galectin-3	Promotion	(37–39)
Core 3	Core 3 synthase	Integrin	Suppression	(40)
O-GlcNAc	OGT	–	Promotion	(51)
Chondroitin sulfate (CS)	–	–	Suppression	(59)
Hyaluronic acid (HA)	HA synthase (HAS1, HAS2, HAS3)	HA receptor (CD44s, CD44v, and RHAMM)	Promotion	(64–70)
GM3	ST3GAL V	CD9	Suppression	(75)

## Author Contributions

YJ and ZX wrote the manuscript. CX, LZ, XS provided language help and writing assistance. SW and DY conceived ideas and modified manuscript. All authors were involved in the conception, preparation of the manuscript, and the final version of the manuscript has been read and approved by all authors before its submission.

## Conflict of Interest

The authors declare that the research was conducted in the absence of any commercial or financial relationships that could be construed as a potential conflict of interest.
